# Satellite glial cells in dorsal root ganglia are activated in streptozotocin-treated rodents

**DOI:** 10.1111/jcmm.12406

**Published:** 2014-10-14

**Authors:** Menachem Hanani, Erez Blum, Shuangmei Liu, Lichao Peng, Shangdong Liang

**Affiliations:** a Laboratory of Experimental Surgery, Hadassah-Hebrew University Medical CenterJerusalem, Israel; b Department of Physiology, Medical School of Nanchang UniversityNanchang, Jiangxi, China

**Keywords:** diabetes, neuropathic pain, satellite glial cells, glial fibrillary acidic protein

## Abstract

Neuropathic pain is a very common complication in diabetes mellitus (DM), and treatment for it is limited. As DM is becoming a global epidemic it is important to understand and treat this problem. The mechanisms of diabetic neuropathic pain are largely obscure. Recent studies have shown that glial cells are important for a variety of neuropathic pain types, and we investigated what are the changes that satellite glial cells (SGCs) in dorsal root ganglia undergo in a DM type 1 model, induced by streptozotocin (STZ) in mice and rats. We carried out immunohistochemical studies to learn about changes in the activation marker glial fibrillary acidic protein (GFAP) in SGCs. We found that after STZ-treatment the number of neurons surrounded with GFAP-positive SGCs in dorsal root ganglia increased 4-fold in mice and 5-fold in rats. Western blotting for GFAP, which was done only on rats because of the larger size of the ganglia, showed an increase of about 2-fold in STZ-treated rats, supporting the immunohistochemical results. These results indicate for the first time that SGCs are activated in rodent models of DM1. As SGC activation appears to contribute to chronic pain, these results suggest that SGCs may participate in the generation and maintenance of diabetic neuropathic pain, and can serve as a potential therapeutic target.

## Introduction

Diabetes mellitus (DM) has become a global epidemic, and with the increasing number of patients there is a greater need to understand and treat the manifold problems associated with this disease. A common complication in DM is neuropathy, which affects 50% of the patients and is associated with several serious problems, one of which is chronic pain [1,2]. The mechanisms of this pain are poorly understood, and therapy is limited [3].

Much of the research on diabetic neuropathy has been done on peripheral mechanisms, and there is strong evidence for major peripheral changes in DM [1,2]. However, there is also considerable evidence for alterations in the central nervous system in animal models for DM. For example in the db/db mouse model, spinal neurons display abnormal biochemical features, and spinal astrocytes are activated [4]. In the STZ model in rats, spinal microglia undergo activation [5,6]. These changes are the results of a combination of the peripheral inflammation, hyperglycaemia and the presence of reactive oxygen species in the spinal cord.

An important station in the pain pathways is sensory ganglia, which contain the somata of sensory neurons and satellite glial cells (SGCs) that tightly surround them [7–13]. Neurons in these ganglia generate abnormal (ectopic) firing in a variety of pain states in humans and in animal models, and thus can contribute to chronic pain [7,14,15]. It has been therefore stated that ‘the single most important factor in the generation and maintenance of chronic neuropathic pain are increased excitability of primary nociceptive afferents’ [14]. Changes in dorsal root ganglia (DRG) neurons in DM models have been assessed in several studies, and have shown that ionic channels in DRG neurons are altered [16,17], suggesting that these ganglia may play a role in diabetic pain. However, there are no reports on possible changes in SGCs in these models.

There is growing interest in the role of glial cells in the central nervous system in chronic pain, and numerous studies showed that in the spinal cord, microglia and astrocytes contribute to the underlying mechanisms [18]. Work in the last decade has demonstrated that SGCs undergo major changes in a large variety of pain models, and that like their central counterparts, they may contribute to chronic pain [9,10,12]. The objective of this work was to test the hypothesis that SGCs are activated in a model of DM1 in mice and rats, and thus may play a role in diabetic neuropathic pain.

## Materials and methods

### Animals

We used C57bl/6 mice, of either sex (M/F ratio 1:1), and male Sprague-Dawley rats. The procedures were approved by the Animal Care and Use Committees of the Hebrew University and Nanchang University Medical Schools. DM type 1 was induced by an intraperitoneal (i.p.) injection of STZ (single 250 mg/kg for mice; high calorie food and a single STZ 65 mg/kg injection for rats). STZ was dissolved in citrate buffer (pH 4.5). Control animals received buffer. After 7 days blood samples were obtained from the tail vein and glycaemia was determined by using a glucometer. Fasting blood glucose >200 mg/dl (11.1 mM) was considered as DM [19,20]. At day 14 the animals were killed by CO_2_ inhalation, and L4,5 DRGs were removed.

### Behavioural study

Individual animals (mice, rats) were placed in a clear plastic box on a wire mesh floor and were allowed to accustom to their new environment for at least 20 min before pain thresholds were assessed by observing withdrawal responses to mechanical stimulation, by using von Frey hairs (Stoelting, Wood Dale, IL, USA). Hairs were applied 10 times at intervals of 5 and 20 s. in ascending order of force. The hairs were pressed against the plantar skin of the hind paw until the hair buckled. Sharp retraction of the stimulated hind paw was considered as a response in the paw. The threshold response was defined when 6 out of 10 responses occurred. Care was taken not to stimulate the same point on the skin in succession.

### Immunohistochemistry

Immunohistochemistry was carried out as follows. Mouse DRGs were removed and placed in 4% paraformaldehyde for 2 h at room temperature. The DRGs were then washed in 0.1 M PBS before incubation in 30% sucrose in PBS overnight before freezing in Tissue-Tek embedding medium (Sakura Finetek, Torrance, CA, USA). Sections were cut 12-μm-thick by using a cryostat (Leica CM 1950; Leica Microsystems, Leitz, Wetzlar, Germany) and thaw mounted on slides. Sections were washed in PBS and incubated in blocking solution containing 3% bovine serum albumin (BSA) in PBS with 0.3% Triton X-100 for 60 min. at room temperature. Primary antibody against GFAP (rabbit anti-GFAP; Dako, Copenhagen, Denmark) was diluted 1:400, in PBS containing 1% BSA and incubated overnight at 4°C. Sections were washed in PBS and incubated with the secondary antibody, donkey anti-rabbit conjugated to DyLight 549-TFP ester (Jackson ImmunoResearch, West Grove, PA, USA) diluted 1:400 in PBS with 10 μmol/l of the fluorescent dye 4′,6-diamidino-2-phenylindole dihydrochloride (DAPI) and 1% BSA for 2 h at room temperature. Finally, sections were washed in PBS. Controls omitted the primary antibody. Sections were imaged by using an upright microscope (Axioskop FS2; Zeiss, Jena, Germany), equipped with fluorescent illumination and a digital camera (Pixera penguin 600CL, Los Gatos, CA, USA). Cells were distinguished by using DAPI, neuronal nuclei are larger and paler compared with SGC nuclei. Neurons that were surrounded by GFAP-positive SGCs by more than 50% of their circumference were counted and expressed as a percentage of the total number of neurons present in the fields analysed; see Warwick and Hanani [21]. Data from each group were collected from four animals. Five randomly selected fields, each containing about 50 neurons were analysed from each animal and averaged. A similar method was used for rat DRGs. Data were analysed by using *t*-test.

### Western blot

Rat thoracic and lumbar DRG were used to maximize the amount of tissue. After isolation ganglia were homogenized by mechanical disruption in lysis buffer and incubated on ice for 40 min. Homogenate was then pelleted at 6,000 *g* for 10 min. and supernatant was collected. By using Lowry method, the quantity of total protein was determined in the supernatant. After being diluted with sample buffer and heated to 95°C for 10 min., samples containing equal amounts of protein (20 μg) were separated by SDS-PAGE by using Bio-Rad system and 12% gel. Protein in gel was transferred onto PVDF membrane by electrophoretic transfer using the same system, the membrane was blocked with 5% non-fat dry milk for 3 h at room temperature, followed by incubation with primary antibody (chicken anti-GFAP 1:1000, Abcam monoclonal β-actin 1:1000; Advanced Immunochemicals, VB Long Beach, CA, USA) overnight at 4°C. The membrane was incubated with secondary antibody, horseradish peroxidase (HRP) conjugated goat anti-rabbit IgG (1:1000; Beijing Zhongshan Biotech, Beijing, China) for 1 h at room temperature. After washing in 25 mM Tris buffered saline, pH 7.2, plus 0.05% Tween 20, chemiluminescent signals were collected on autoradiography film by using enhanced chemiluminescence kit (Shanghai Pufei Biotech, Shanghai, China). Band intensities were measured by using Image-Pro Plus software (Media Cybernetics company, USA) and normalized to each β-actin internal control. Statistical analysis of the data was performed on SPSS 11.5 software, Chicago, Illinois, USA. All the results were expressed as mean ± SD from at least three independent trials. Six animals were included in each group. Statistical significance was determined by unpaired *t*-test. *P* < 0.05 was considered as statistically significant.

## Results

**Fig. 1:**
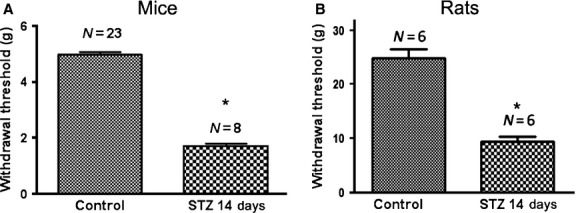
Streptozotocin (STZ) injections cause tactile hypersensitivity in mice and rats. Withdrawal responses were measured in the hind paws with von Frey hairs. The pain threshold in 14 days after STZ administration is significantly lower than in control animals, *P < 0.05. The numbers above the bars indicate the number of animals tested.

Only animals in which STZ treatment elevated fasting blood glucose above 200 mg/100 ml were used for the following studies. The values (in mg/100 ml) at 14 days after STZ injection were for mice 97.8 ± 2.0 in controls, and 376.8 ± 18.8 after STZ treatment (*N* = 4 for each group). For rats the values were 82.9 ± 11.59 in controls and 430.0 ± 68.0 (*N* = 6 for each group). The STZ-treated animals showed reduced threshold for mechanical stimulation (Fig. 1), consistent with the presence of DM-like neuropathic pain.

**Fig. 2:**
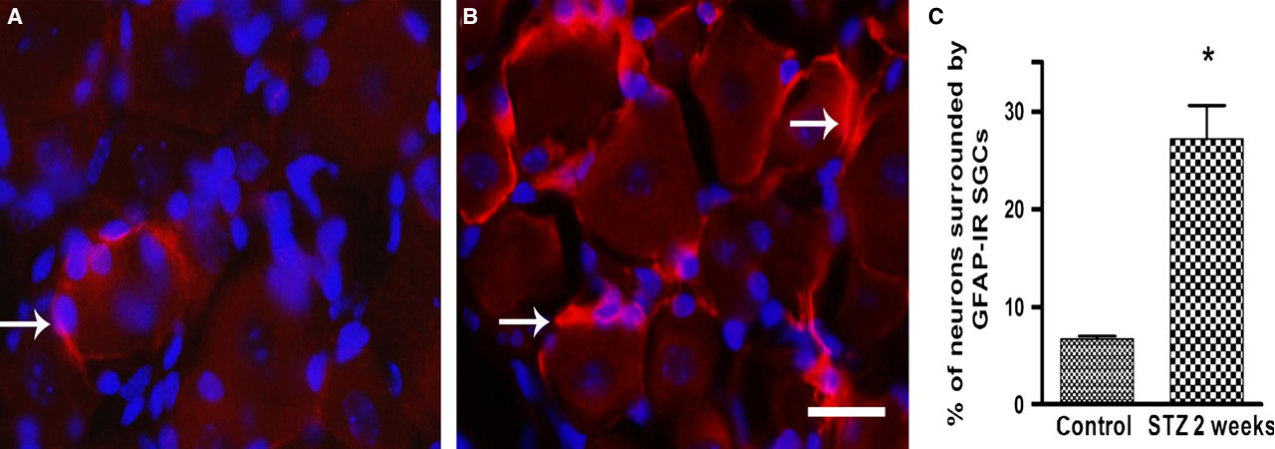
GFAP was up-regulated in SGCs in mouse DRG 14 days after STZ injection. (**A**) Control (**B**) Treated animals. Note that many neurons are surrounded with GFAP-positive SGCs. The two tissues were processed and imaged under the same conditions. Calibration bar, 20 μm. (**C**) Quantitation of GFAP expression in SGCs in DRG from STZ treated mice. Data for the control group and the STZ treated group were collected from 4 mice each. The asterisk indicates *P* < 0.05 compared with control, error bars indicate SD.

**Fig. 3:**
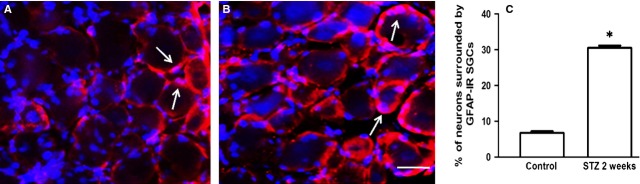
GFAP was up-regulated in SGCs in rat DRG 14 days after STZ injection. (**A**) Control (**B**) Treated animals. Note that many neurons are surrounded with GFAP-positive SGCs. The two tissues were processed and imaged under the same conditions. Calibration bar, 20 μm. (**C**) Quantitation of GFAP expression in SGCs in DRG from STZ treated rats. Data for the control group and the STZ treated group were collected from 4 rats each. The asterisk indicates *P* < 0.05 compared with control, error bars indicate SD.

Fourteen days after STZ administration there was a 4-fold increase in the number of neurons surrounded by a ring of GFAP-immunopositive SGCs in ganglia from mice (Fig. 2A and B). Figure 2C shows a quantitative analysis of the data. Similar results were obtained in rats (Fig. 3), where the number of neurons surrounded with GFAP-IR SGCs in DRG from STZ-treated rats increased by 4-fold compared with control rats. In both cases, *P* < 0.05.

**Fig. 4:**
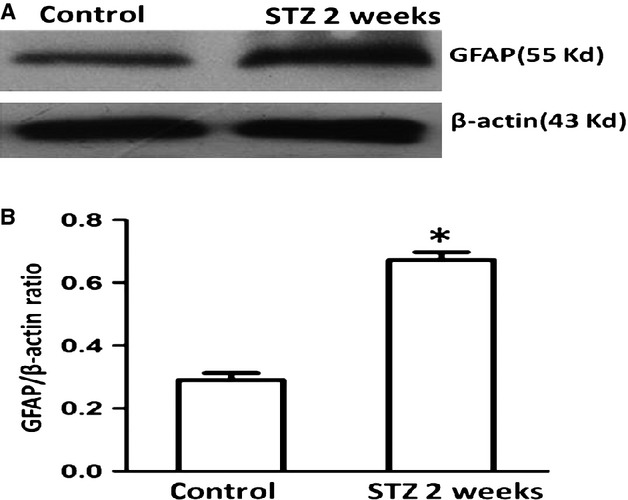
GFAP was up-regulated in rat DRG 14 days after STZ injection, as demonstrated by Western blot study. GFAP protein expression in STZ-treated rats was significantly increased in comparison with that in control rats. *N* = 6 for each of the groups. The asterisk indicates *P* < 0.05 compared with control, error bars indicate SD.

To obtain quantitative information on the changes in GFAP in STZ-treated rats, we carried out a Western blot study. As shown in Figure 4, there was an over 2-fold increase in the amount of GFAP protein, in accord with the immunostaining results.

## Discussion

Neuropathy and pain are common in DM patients, but the underlying mechanisms have received relatively little attention. We show here for the first time that SGCs in DRG in the STZ model are activated and therefore could contribute to pain in DM. The observations were done on both mice and rats, which help to generalize the conclusions.

The activation of SGCs described here might be secondary to damage to peripheral nerves, which is known to occur in both humans and animals with DM [1]. It was reported that the augmented firing in the axons of DRG neurons following peripheral damage is an important factor in SGCs activation as assayed by GFAP immunohistochemistry [22]. However, how neuronal firing induces GFAP up-regulation is obscure. It is possible that high glucose and other pathologies in DM have a direct effect on SGCs. It was found that increased pain responses start 5 days after STZ injection [23], whereas decreased conduction velocity, which is an accepted measure for nerve damage, was detected only at 14 days [24]. Thus, it is conceivable that the changes in SGCs are, at least partly, independent from the changes in the nerves.

We used mice for this work because this species has become the animal of choice in biomedical research because of the numerous mutants and knockouts mice that are available. We decided to use also rats because rats are still the most frequently used animals in diabetes and pain research, and DRG in these animals have been under extensive investigation for decades. In addition, for biochemical studies rats are usually preferable because larger amounts of tissue can be obtained from them in comparison to mice.

The results for mice and rats were very similar, and in both species STZ administration caused a significant increase in GFAP staining. As the DRG in rats are much larger than in mice, we carried out Western blotting on them, and found that the amount of GFAP was doubled in DM1 model. This similarity in the results adds to the validity of the results and indicates that are likely to hold in other species.

GFAP augmentation is a marker for glial activation, which is accompanied by a variety of pathological changes in these cells. For example, in mouse and rat pain models, activation is associated with a large increase in the number of gap junctions connecting SGCs around different neurons [25–27], and augmented responses to the pain mediator ATP [28]. Other investigators have observed increased cytokine production [13,29] in SGCs in pain models. We therefore propose that SGCs activation in the DM1 models is associated with other changes that may have functional significance, and may contribute to abnormal neuronal activity, and to behavioural effects.
